# A cross sectional survey of knowledge, attitude and practices related to house flies among dairy farmers in Punjab, Pakistan

**DOI:** 10.1186/1746-4269-9-18

**Published:** 2013-03-19

**Authors:** Hafiz Azhar Ali Khan, Waseem Akram, Sarfraz Ali Shad, Muhammad Razaq, Unsar Naeem-Ullah, Khuram Zia

**Affiliations:** 1 Department of Entomology, Bahauddin Zakariya University, Multan, Pakistan; 2 Institute of Agricultural Sciences, University of the Punjab, Lahore, Pakistan; 3 Department of Agri. Entomology, University of Agriculture, Faisalabad, Pakistan

## Abstract

**Background:**

House flies are of major public health concerns in areas with poor sanitation and hygienic conditions. Unfortunately, sanitation and hygiene have always been ignored in dairy farms particularly in the developing or low-income countries. Management of these flies mostly depends on the awareness regarding associated hazards and protective measures taken by the people to minimize risks associated with flies. The present study therefore explores the knowledge, attitude and practices taken by dairy farmers in Punjab, Pakistan against house flies.

**Methods:**

The present study was based on a cross sectional self administered survey to a convenience sample of 173 small scale dairy farmers in four localities – Multan, Lahore, Shorkot and Faisalabad – of Pakistan. The relationships between socio-demographics, knowledge and preventive practices were investigated through logistic regression analysis and chi-square test of association.

**Results:**

Considerable number of dairy farmers 71/173 (41.04%) had no idea about the problems associated with house flies. Although 77/173 (44.51%) dairy farmers reported house flies as disease transmitters, only 23 (29.87%) farmers were familiar with diseases and 22 (28.57%) had somewhat idea of the mode of disease transmission. We found a positive association between dairy farmer’s education level and overall knowledge of house flies in multivariate analysis. Farmer’s education level and knowledge of the house flies breeding sites had a positive association with the adoption of house fly prevention practices by the respondents. However, knowledge of the problems associated with house flies and preventive measures had no association with house fly prevention practices.

**Conclusion:**

The present ethnoentomological survey provides information about knowledge, attitude and practices of dairy farmers related to house flies in Punjab, Pakistan. We conclude that the farmers’ education level and knowledge of the breeding sites had a positive association with the adoption of prevention practices against house flies. The study also highlights the need of targeting the lack of knowledge of dairy farmers for the successful management of house flies.

## Background

Human being’s perception about natural world largely influences the way one thinks and behaves with animals and plants. The attitude towards animals depends on humans knowledge, perception, and nature of the relationship they have with a particular animal [1]. For example, Mofu people in Africa have a peculiar type of behaviors towards insects, ants and termites in particular, of their environment [2]. A *Dorylus* sp. of ant, known as “Jaglavak” in Africa, is considered as the prince of the insect fauna. Whenever they find it, they treat it with respect and fear. Generally, they call it *Bi* (boss), and bend over and touch their chest. The analysis of knowledge, beliefs, thoughts and uses of insects by human culture in ethnoentomological studies provides us an opportunity to better understand the life of ethnic group being studied [1].

The house fly, *Musca domestica* L. (Insecta: Diptera: Muscidae), has been considered as a notorious pest by medical, veterinary and public health professionals worldwide. The synanthropic nature of house flies has made them a potential pest for spreading various diseases from man to man and/or animals to man. More than 100 animal diseases have experimentally been associated with house flies including protozoan, bacterial, viral and helminthic infections [3]. Outbreaks of intestinal diseases like diarrhea in urban and rural settlements, including Pakistan [4] are closely related to the seasonal abundance of house flies while their control results in the decline of such outbreaks [5]. According to recent reports, house flies are also known as potential carrier of avian influenza or bird flu virus, posing periodic threats to humans and poultry industry worldwide [6]. In Pakistan, this disease was reported in 1995 [5], since then various periodic outbreaks have been reported in different parts of the country. There is a possibility of recurrence of avian influenza outbreaks in Pakistan because vaccination against the virus is not rigorously practiced [7]. Moreover, the practice of intensive animal farming coupled with high temperature, humidity, lack of sanitation and quantities of manure provides ideal environments for the development and rapid expansion of house fly populations [5]. This expansion of flies in animal units could cause future outbreaks of avian influenza and other diseases in the country. Besides avian influenza, diarrhea is another disease and has been considered a leading cause of childhood death in Pakistan [4]. In Pakistan, according to an estimate, every child under the age of 5 years is estimated to suffer on an average 5 episodes of diarrhea per annum. Previously house flies have been reported as a major cause of childhood diarrhea in Pakistan and it was suggested that fly control measures could help to reduce the incidence of diarrhea [8].

House flies are of major public health concerns in areas with poor sanitation and hygienic conditions [9]. Pakistan is among the developing countries and has issues related to sanitation and hygiene that have been given low priority in developing or low-income countries in comparison to other development needs. Being an agricultural country, dairy farming plays a pivotal role in Pakistan’s economy by providing milk, meat, wool, hide, blood, bones and farm animal manures [10]. The dairies usually play a significant role in the breeding of flies due to poor hygiene conditions in the form of farm manures, poor disposal and open defecation places. All such places are recognized as potential feeding and breeding places of house flies [11]. Improving environmental sanitation and hygiene of dairies, for example reduction of the breeding sites and sources that attract flies, provide effective house fly control. Moreover, the installation and use of latrines with a concealed sewage system in such places can further minimize various diseases spread due to the inability of flies to come in contact with feces and pathogens [12]. Therefore, there is a dire need to educate dairy farmers about the hazards of flies, hygiene practices and how to best manage their farm animal waste to minimize fly breeding. However, such health education and hygiene promotion campaigns can only be successful if they are based on the current knowledge of the target communities [4]. Keeping in view the economic importance of house flies, the present study was designed with the objective to assess knowledge, attitude and practices (KAP) of dairy farmers in relation to house fly’s hazards and their management. In the present cross sectional survey, a stepwise approach was adopted to check the following research hypotheses: a) overall knowledge of house flies among dairy farmers vary with the socio-demographic status of the farmers particularly the education level, and b) the adoption of preventive measures to get rid of house flies depends on the education level and knowledge of the farmers about house flies’ hazards, breeding places and preventive measures. To date, such KAP studies have been rare in the house fly research. However, the results presented in this study can help to develop appropriate policy decisions aimed at management of house flies by improving the knowledge and attitude of dairy farmers, and sanitation, hygiene and farm animal waste management practices.

## Methods

### Study sites and ethnographic background

The study was carried out in rural areas of four localities of Punjab, Pakistan: Multan (30° 11' 44 N; 71° 28' 31 E), Shorkot (30° 46' 60 N; 72° 15' 0 E), Lahore (31° 32' 59 N; 74° 20' 37 E) and Faisalabad (31° 26' 0" N; 73° 6' 0 E). Punjab is the most populated province of Pakistan, which accommodates almost 50% country’s population and is the major milk-producing province of the country. It is situated at the northwestern edge of the geologic plate in South-Asia. The study areas have three major seasons [13]: hot season when the mercury rises as high as 110°F (April to June), Rainy season with an average annual rainfall of 46 cm in the plains (July to September), and mild season with the mercury goes down as low as 40°F. Dairy, poultry and crop farming are the major economic activities of local people. Among crop farming, cotton, wheat, rice, maize, pulses, oilseeds, vegetables and fruits like citrus and mango, are important crops of the region.

Dairy sector in Punjab, Pakistan has almost similar characteristics like that of other developing Asian nations e.g., small herd, low quality feeds, poor genetic potential of animals, frequent epidemics, lack of technical skills etc. The majority of dairy farmers (70%) in Pakistan are smallholding farmers, which contribute 90% in the country’s milk production. The international farm comparison network (IFCN) has categorized the small scale farmers of Pakistan into four major categories based on area of land holdings and the number of milk animals [14]. The PK-3 category (a farmer has at least 3 ha land and 3 milk animals) was selected for study purpose since it contributes nearly 70% of the small scale farms and have a significant impact on the country’s economy. Moreover, the growth of these small scale farms is very poor due to many factors; of these unhygienic conditions are the major constraints, which need to be regulated.

### Study design

A KAP survey questionnaire was developed keeping in view the guidelines of Frary [15]. The questionnaire consisted of three main parts for collection of data on socio-demographic characteristics of dairy farmers, their knowledge about house flies and associated problems, and farmers’ practices for the management of house flies. Both open and closed ended questions were included in the questionnaire. The localities and dairy farmers were selected based on convenience sampling, and availability of farmers on study day and their willingness to participate. Knowledge of house flies was assessed by asking questions related to disease spread, mode of disease transmission, high breeding times, breeding places and preventive measures. Verbal consent from the farmers was obtained prior to the interviews and the Board of Advanced Studies Committee of Bahauddin Zakariya University, Multan, ethically approved the study protocol.

### Data management

The data on farmers’ knowledge and practices was scored by following the methodology of Koenraadt et al. [16]. Briefly, knowledge of the problems associated with the house fly was defined by the interviewee mentioning at least one of the following problems: nuisance, disease vector, interference in the milking process (Score = 1, if he/she mentioned at least one or more problems; and score = 0, if he/she failed to mention any problem). A farmer was considered knowldgeful about house flies breeding sites if he/she mentioned at least one of the following breeding sites: filth, farm animal manure, human excreta, spilled feed, meat, decaying organic matter (Score = 1, if he/she mentioned at least one or more breeding sites; and score = 0, if he/she failed to mention any breeding site). Similarly, a farmer had the knowledge of house flies preventive measures if he/she mentioned at least any one of the following: sanitation, door or window screening, fly papers, insecticides, covered storage of the farm yard manure (Score = 1, if he/she mentioned at least one or more preventive measures; and score = 0, if he/she failed to mention any preventive measure). Overall knowledge of house flies was defined as the sum of the score of: knowledge of the problems associated with house flies (0/1), knowledge of the house flies’ breeding sites (0/1) and knowledge of the preventive measures (0/1). Resultantly each farmer obtained a score 0 to 3. The house fly prevention practices were defined by the farmers using at least one of the following preventive measures: screening of windows or doors, sanitation, insecticides, repellents, safe storage of farm yard manure.

### Statistical analyses

The data were analyzed by chi- square test of association, binary logistic and ordinal logistic regression models using SPSS software (Version 10.0 for windows, SPSS Inc., Chicago, USA). Multivariate logistic regression analyses were performed separately to determine the predictors of house fly knowledge, and adoption of management practices at farm level. For these, univariate analyses were performed first, and the potential predictors having screening significance P < 0.25 were then entered in the initial multivariate model [17]. In succeeding steps, the predictors with a P > 0.05 in previous step were removed from the model until complete loss of fit of the model was achieved.

## Results

### Socio-demographic characteristics and farm waste management practices

**Figure 1:**
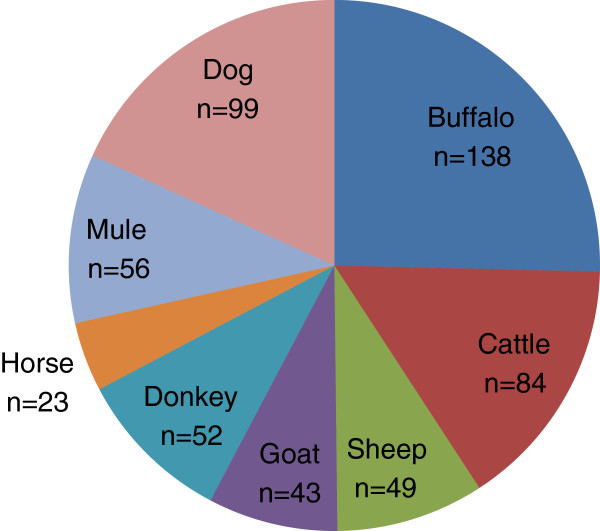
**Types of animals being reared by the respondents.** n = number of farmers domesticating particular type of animals. Total number exceeds 173 due to multiple animal farming by the respondents.

The socio-demographic characteristics of the respondents are presented in Table 1. In total, 173 dairy farmers from four localities of the Punjab province, Pakistan were interviewed. Most of the respondents were male as females were reluctant to give interviews because of religious values and/or conservative style. The education level of the farmers revealed that most of them were illiterate, while a small fraction had their education up to secondary or graduate level. To examine whether there were significant differences in the socio-demographic characteristics among the farmers in samples across the four research locations or not, a chi-square test was conducted. There were no significant differences in socio-demographics among the respondents from all the localities of Punjab (P > 0.05) (Table 1). Most of the farmers were engaged in multiple animal farming systems (Figure 1). Cattle, buffalo, sheep and goat were the major animals being reared for dairy purpose. Donkey, horse and mule were the major equine mainly used for draught and transportation purposes while dogs were being reared for security purpose. Most of the respondents were also engaged in crop farming in addition to dairy farming.

**Table 1:** Socio-demographic characteristics of dairy farmers in four localities of the province Punjab, Pakistan (n = 173)

**Socio-demographic characteristics**	**Multan**	**Lahore**	**Faisalabad**	**Shorkot**	**Total**
	**n (%)**	**n (%)**	**n (%)**	**n (%)**	**n (%)**
**Gender**					
Male	41 (78.85)	43 (86.00)	35 (81.40)	20 (71.43)	139 (80.35)
Female	11 (21.15)	7 (14.00)	8 (18.60)	8 (28.57)	34 (19.65)
**Age (year)**					
≤ 25 years	11 (21.15)	11 (22.00)	11 (25.58)	9 (32.14)	42 (24.28)
26-39	23 (44.23)	23 (46.00)	15 (34.88)	9 (32.14)	70 (40.46)
≥ 40	18 (34.62)	16 (32.00)	17 (39.53)	10 (35.71)	61 (35.26)
**Education level**					
Illiterate	23 (44.23)	26 (52.00)	21 (48.84)	10 (35.71)	80 (46.24)
≥ Primary	18 (34.62)	11 (22.00)	14 (32.56)	13 (46.43)	56 (32.37)
≥ Secondary	8 (15.38)	9 (18.00)	7 (16.28)	3 (10.71)	27 (15.61)
≥ Graduation	3 (5.77)	4 (8.00)	1 (2.33)	2 (7.14)	10 (5.78)
**Marital status**					
Single	6 (11.54)	8 (16.00)	13 (30.23)	7 (25.00)	34 (19.65)
Married	43 (82.69)	41 (82.00)	28 (65.12)	20 (71.43)	132 (76.30)
Widow/widower	3 (5.77)	1 (2.00)	2 (4.65)	1 (3.57)	7 (4.05)

Almost all the respondents either stored the farm animal waste and manure in open fields or threw it out. The majority of the respondents did not have latrines in their compound, and they usually used to defecate in open fields (Table 2).

**Table 2:** Farm yard manures management and defecation practices of dairy farmers (n = 173)

	**n**	**Percent**
People throw farm yard manure in open fields	93	53.76
People store manure in open fields	170	98.27
Latrine available in the compound	54	31.21
People defecate in open fields	123	71.10
People defecate in the latrine	50	28.90

### Knowledge of problems associated with house flies

Of the 173 respondents who had ever heard about house flies, 102 dairy farmers could mention at least one problem associated with house flies (Table 3). The most commonly mentioned problem was nuisance followed by disease transmission in humans and farm animals. The respondents (n = 77) who declared the house fly as a disease transmitter, were further questioned about the diseases and mode of disease transmission by the house fly. Avian influenza/bird flu, a deadly disease of humans and birds vectored by the house fly, was mentioned by only one person while diarrhea was mentioned by 22 respondents. Numerous respondents didn’t name any disease transmitted by the house fly. Similarly, most of the respondents were unaware about the mode of disease transmission by house flies (Table 3).

**Table 3:** Knowledge of problems associated with house flies, mode of disease transmission, potential breeding season and places

	**Multan**	**Lahore**	**Faisalabad**	**Shorkot**	**Total**
	**n (%)**	**n (%)**	**n (%)**	**n (%)**	**n (%)**
**Have you heard about house flies?**					
-Yes	52 (100%)	50 (100%)	43 (100%)	28 (100%)	173 (100%)
-No	0	0	0	0	0
**Knowledge of the problems associated with house flies**					
-These cause nuisance to humans	13 (25.00)	13 (26.00)	9 (20.93)	6 (21.43)	41 (23.70)
-These cause nuisance to farm animals	1 (1.92)	3 (6.00)	2 (4.65)	1 (3.57)	7 (4.05)
-These interfere the milking process	4 (7.69)	4 (8.00)	2 (4.65)	2 (7.14)	12 (6.94)
-These transmit diseases in humans	8 (15.38)	8 (16.00)	6 (13.95)	6 (21.43)	28 (16.18)
-These transmit diseases in farm animals	5 (9.62)	7 (14.00)	6 (13.95)	1 (3.57)	19 (10.98)
-These transmit diseases both in humans and animals	9 (17.31)	9 (18.00)	6 (13.95)	6 (21.42)	30 (17.34)
I don’t know	20 (38.46)	19 (38.00)	20 (46.51)	12 (42.86)	71 (41.04)
Knowledge of the diseases transmitted by house flies*					
House flies cause Avian influenza/bird flu	1 (4.00)	0 (0.00)	0 (0.00)	0 (0.00)	1 (2.60)
House flies cause diarrhea	8 (32.00)	8 (36.36)	5 (27.78)	1 (8.33)	22 (28.57)
I don’t know	17 (68.00)	14 (63.63)	13 (72.22)	11 (91.66)	55 (71.43)
Mode of disease transmission by house flies*					
-These contaminate food and drinking water	3 (11.54)	2 (9.09)	1 (5.56)	1 (9.09)	7 (9.09)
-These cause Infection through wounds	5 (19.23)	6 (27.27)	4 (22.22)	1 (9.09)	16 (20.78)
I don’t know	18 (69.23)	16 (72.72)	11 (61.11)	10 (90.91)	55 (71.43)
**Usual active time of house flies**					
-These remain active at day time	26 (50.00)	25 (50.00)	22 (51.16)	18 (64.29)	91 (52.60)
-These remain active at night time	5 (9.62)	2 (4.00)	3 (6.98)	0 (0.00)	10 (5.78)
-These remain active every time	21 (40.38)	23 (46.00)	18 (41.86)	10 (35.71)	72 (41.62)
**Knowledge of the house flies breeding season**					
**House flies breed more in**					
-Summer	17 (32.69)	17 (34.00)	14 (32.56)	10 (35.71)	58 (33.53)
-Winter	6 (11.54)	9 (18.00)	3 (6.98)	3 (10.71)	21 (12.14)
-Spring	8 (15.38)	10 (20.00)	4 (9.30)	1 (3.57)	23 (13.29)
I don’t know	22 (42.31)	15 (30.00)	24 (55.81)	14 (50.00)	75 (43.35)
**Knowledge of the house flies breeding places**					
**House flies breed in**					
-filth of any type	18 (34.62)	12 (24.00)	11 (25.58)	10 (35.71)	51 (29.48)
-human excreta	13 (25.00)	12 (24.00)	13 (30.23)	8 (28.57)	46 (26.59)
-farm animals’ manure	2 (3.85)	1 (2.00)	1 (2.33)	1 (3.57)	5 (2.89)
I don’t know	25 (48.08)	29 (58.00)	21 (48.84)	12 (42.86)	87 (50.29)

*Response of respondents who answered the house fly as a disease transmitter (n = 77).

Total percentage of most of the categories exceed that of 100% due to multiple responses.

For the usual active time of house flies, 52.60% thought that these remain active during the day time, whereas 41.62% said every time whether day or night. Approximately, one third of the respondents said that summer is the more intense breeding season of flies followed by spring and winter. Filth of any type and human excreta were the breeding places of house flies, which were referenced most often whereas half of the respondents had no knowledge about the breeding places. There were no significant differences about the knowledge of the problems associated with house flies, their active period, potential breeding seasons and places among the respondents from four localities (P > 0.05) (Table 3).

### Knowledge of preventive measures

According to the interviewee, 38.15% thought that house flies were preventable (Table 4). Knowledge of using plants to control or prevent house flies was cited by 48 farmers. Of these, most of the farmers said that house flies could be controlled by using neem (*Azadirechta indica*) essential oils, while some farmers were in the opinion that house flies could be deterred by planting mint plants (*Mentha* spp.) around the living facilities. According to the interviewee, these two plants were easy to plant and their specific fragrances deter many pests. Sanitation and use of electric fly killers as tools to prevent house flies were also cited by most of the farmers whereas a small fraction answered “I don’t know any measure”.

**Table 4:** Knowledge of the respondents about the prevention measures for house flies

	**Multan**	**Lahore**	**Faisalabad**	**Shorkot**	**Total**
	**n (%)**	**n (%)**	**n (%)**	**n (%)**	**n (%)**
**Are house flies preventable?**					
-Yes	20 (38.46)	21 (42.00)	13 (30.23)	12 (42.86)	66 (38.15)
-No	5 (9.62)	5 (10.00)	5 (11.63)	2 (7.14)	17 (9.83)
I don’t know	27 (51.92)	24 (48.00)	25 (58.14)	14 (50.00)	90 (52.02)
**Knowledge about preventive/control measures**					
**House flies can be controlled/prevented by**					
-using fly papers	1 (1.92)	2 (4.00)	0 (0.00)	0 (0.00)	3 (1.73)
-using insecticides	6 (11.54)	3 (6.00)	3 (6.98)	2 (7.14)	14 (8.09)
-sanitation	16 (30.77)	16 (32.00)	11 (25.58)	8 (28.57)	51 (29.48)
-using fans	6 (11.54)	3 (6.00)	1 (6.98)	1 (3.57)	11 (6.36)
-planting mint (*Mentha* spp.)	4 (7.69)	2 (4)	1 (2.33)	2 (7.14)	9 (5.20)
-using neem (*Azadirachta indica*) essential oil	15 (28.85)	9 (18)	7 (16.28)	8 (28.57)	39 (22.54)
-using electric fly killer	11 (21.15)	15 (30)	17 (39.53)	8 (28.57)	51 (29.48)
I don’t know any measure	4 (7.69)	6	4 (7.69)	0	14 (8.09)

Total percentage of most of the categories exceed that of 100% due to multiple responses.

**Figure 2:**
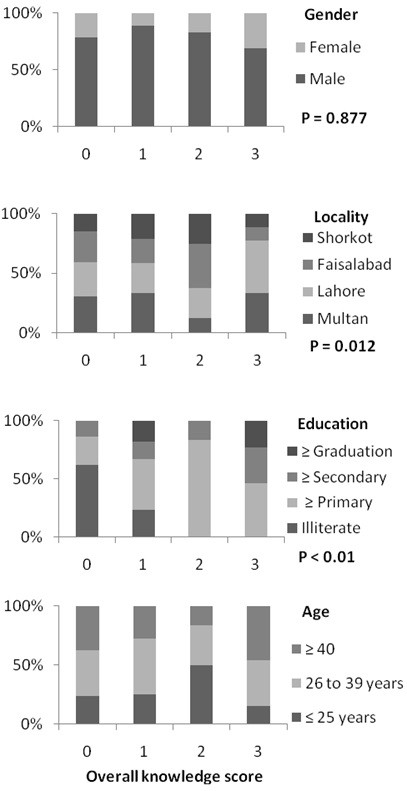
**Determinants of overall house fly knowledge in the province of Punjab, Pakistan.** Overall knowledge of house flies was defined as the sum of the score of knowledge of the problems associated with house flies, knowledge of house flies’ breeding sites and knowledge of preventive measures. Resultantly each farmer obtained a score between 0 to 3. Probability values are based on univariate ordinal regression model and reveal the overall impact on differences in knowledge scores between the categories of each determinant.

Determinants of house fly knowledge are presented in Figure 2. Education level and localities were significantly related with overall knowledge of house flies in univariate analysis. When age, localities and education (all had screening significance; P < 0.25) were entered in multivariate analysis, localities and education level of dairy farmers had significant association with overall knowledge of the house fly (Table 5).

**Table 5:** Ordinal logistic regression models of socio-demographics and knowledge of the house fly

**Model**	**H**_**0**_	−2LL*	***P*£**
Locality + Age + Education + Int**		142.76	---
Locality + Age + Education	β_Int_ = 0	146.83	0.250
Locality + Education	β_Age_ = 0	151.11	0.015

*-2 log likelihood, ** All possible 2 way interactions, **£** p values based chi square of −2 log likelihood difference between the reduced model and initial model. The predictors: localities, age and education level of the farmers had P values < 0.25 and were the potential predictors in univariate analysis (Figure 2). These three potential predictors were then entered in the multivariate model by following the methodology of Hosmer and Lemeshow [17]. In succeeding steps, the predictors with a P > 0.05 in the previous step were removed from the model until complete loss of fit (P < 0.05) of the model was achieved.

Three knowledge determinants viz., education, knowledge of breeding sites and knowledge of preventive measures were significantly related to the house fly prevention practices (P < 0.05) being adopted by the respondents in 0.42, 0.27 and 4.42 odds ratios, respectively (Table 6). However, when the education level and knowledge of breeding sites were included in a multivariate regression model together with the localities and knowledge of the preventive measures (all had screening significance; P < 0.25), the farmers with knowledge of at least one breeding site and higher education level had significant effect on house flies prevention practices (Table 7).

**Table 6:** Effect of socio-demographic characters and house fly knowledge on the use of preventive measures against house flies

**Variables**	**n**	**Percentage of respondents using ≥ 1 preventive measure**	**Odds ratio**	**95% CL**	**P**
**Locality**					
Multan	52	46.15			
Lahore	50	30.00			
Faisalabad	43	23.26			
Shorkot	28	35.71	1.39	0.97-1.99	0.075
**Gender**					
Male	139	35.25			
Female	34	29.41	1.08	0.38-3.06	0.878
**Age (year)**					
≤ 25 years	42	47.62			
26-39	70	28.99			
≥ 40	61	31.14	1.08	0.61-1.92	0.798
**Education**					
Illiterate	80	13.75			
≥ primary	56	47.27			
≥ secondary	27	55.56			
≥ graduation	10	70.00	0.42	0.25-0.71	0.001
**Knowledge of the problems associated with house flies**					
No problem mentioned	132	30.30			
At least one	41	46.34	1.22	0.44-3.40	0.760
**Knowledge of the house flies breeding sites**					
No breeding site mentioned	87	16.09			
At least one breeding site mentioned	86	52.33	0.27	0.11-0.66	0.004
**Knowledge of preventive measures**					
No preventive measure mentioned	14	42.00			
At least one preventive measure mentioned	159	33.33	4.42	1.14-17.13	0.032
**General house fly knowledge scores**					
0	118	34.75			
1	36	33.00			
2	6	50.00			
3	13	23.08	1.12	0.72-1.75	0.605

**Table 7:** Multivariate logistic regression models of the impact of socio-demographics and house flies knowledge on house flies preventive measures

**Model**	**H**_**0**_	−2LL*	***P*£**
Locality + Education + Breeding site knowledge + Preventive measures knowledge + Int**		70.51	----
Locality + Education + Breeding site knowledge + Preventive measures knowledge	β_Int_ = 0	71.78	0.866
Education + Breeding site knowledge + Preventive measures knowledge	β_Locality_ = 0	74.74	0.261
Education + Breeding site knowledge	β_Preventive measures knowledge_ = 0	76.76	0.043

*-2 log likelihood, ** All possible 2 way interactions, **£** p values based on chi square of −2 log likelihood difference between the reduced model and initial model. The predictors: locality, education level of the farmers, farmers’ knowledge about house flies breeding sites and preventive measures, had P values < 0.25 and were the potential predictors in univariate analysis (Table 6). These four potential predictors were then entered in the multivariate model by following the methodology of Hosmer and Lemeshow [17]. In succeeding steps, the predictors with a P > 0.05 in the previous step were removed from the model until complete loss of fit (P < 0.05) of the model was achieved.

In order to develop chemical based management strategies for house flies in the future, we investigated the farmers who used chemical measures for house flies management. Although there were many farmers who were using chemical measures for different dairy and field crop pests, a few farmers had experience of using chemicals directly against house flies or dairy pests. The insecticides from all the classes were in use while farmers were using self-experiences in the choice and use of insecticides.

## Discussion

The analysis of knowledge, beliefs, thoughts and uses of insects by human culture in ethnoentomological studies provides us an opportunity to better understand the life of ethnic group being studied. In terms of ethnoentomological studies, Pakistan is a blank state on the quilt of global research. Although house flies are major public health and veterinary pests, little information is available on the relationship between humans and different aspects of house flies. In the present study, knowledge and perceptions about the house fly, its associated problems, mode of disease transmission, and prevention measures in dairy farming communities were assessed. Understanding local knowledge in the field of ethnobiology could be a strong tool in community health. Since biomedical healthcare, both in quality and quantity, is usually lacking in poor communities, local knowledge could fill the gap between the need of health facilities and their provision *in situ*[18]. For example, Panghal et al. [19] demonstrated the importance of indigenous plant knowledge by the people of Haryana, India, by showing that the communities who had better knowledge of plants also had better management of snakebites.

Our results indicate that the respondents from the four localities in Punjab, Pakistan did not have sufficient knowledge on problems associated with house flies despite the fact of being very common pest in urban and rural settlements. Although some farmers were aware that house flies could transmit diseases in animals, only a small fraction had the knowledge of specific diseases and their transmission mode. Despite the fact of a large number of diseases transmitted by house flies [6,12,20], only the two were mentioned which revealed very poor knowledge of the farming community. Lack of disease knowledge with the majority of the subjects is a matter of concern for the adoption of preventive measures. There was also a lack of knowledge about mode of disease transmission. Even this knowledge at the community level would be encouraging because preventive measures against the house fly might remain the same (proper removal of human and animal excreta, sanitation etc.). It is important for farming communities to be aware of these facts.

The awareness of house flies’ breeding sites, active time and preventive tools are essential components to reduce chances of the house fly-human or house fly-food contacts. In the present study, filth of all types and human excreta as common breeding places were known to some of the respondents. Whereas, sanitation and the use of specific plants were cited by most of the respondents as preventive tools. At the farm level, house flies could be controlled effectively by the reduction and/or elimination of breeding habitats [11,12]. Farm animals and animal manures are an important part of the rural environment and both cannot be segregated. Resultantly, animal manures provide ideal habitats for fly breeding; therefore, there is a dire need to educate dairy farmers about best management practices for farmyard manures so that fly breeding could be minimized. In ethnobotanical context, two plants, *Mentha* spp. and *A. indica*, were cited by the respondents as preventive tools against house flies. Although synthetic insecticides could help to manage house flies, some side effects to the environment and development of insecticide resistance in house flies [21,22] focus on the need to explore alternative insecticides. *A. indica* essential oils have insecticidal potential and used previously to manage house flies and other pests in different parts of the world [23]. Sound knowledge of plants in community health could be very helpful particularly in cases where mainstream health care is often lacking. For example, Gonzalez et al. [24] reported the importance of indigenous plant knowledge by showing that the traditional plant knowledge could be very effective in the management of vectors of emerging diseases (e.g., flies and mosquitoes) without harming the environment. Therefore, there is a need to update the ethnobotanical knowledge of local people in relation to environment friendly techniques for house flies management.

Almost all the respondents either stored farm manure in open fields or threw it in open fields. The majority of the respondents did not have latrines in their compound and they usually used to defecate in open fields. The manure and human excreta in open environment may provide conducive breeding places for the expansion of house flies and could result in the future epidemics of different diseases. Although the level of knowledge and perception about house flies was moderate among dairy farmers, we have little evidence that this knowledge was brought into practice in terms of preventive measures. The results revealed that the education level and knowledge of the breeding sites in our study had a positive and significant effect on the adoption of prevention practices; whereas knowledge of the problems associated with house flies and preventive measures had no effect on house flies prevention measures. The results clearly indicate weak associations between overall house fly knowledge score and adoption of preventive measures. Having a good knowledge about a particular subject does not necessarily lead to practice, since it is difficult to change the behavior of a person [16]. Since little information related to ethnobiology in general and ethnoentomology in particular is being delivered to the Pakistani school students through their school curricula, the students or the communities are hard to be cautious about different insect pests or insect borne diseases [personal observation]. Recently, a little information about dengue fever and dengue mosquito has been added in school curricula after the occurrence of a severe epidemic in Punjab, in 2011. The incorporation of ethnobiological knowledge, thoughts and practices in school curricula, both in the form of folksongs or storytelling, can be helpful for the well being of developing generation and can put scientific learning within its traditional context [25,26]

### Limitations

The use of convenience sampling makes the generalisability of the study limited. The sample was drawn from small scale farmers only, the findings may not be generalisable to all categories of farmers defined by IFCN. However, since dairy farms were not specified in a particular area [10] and considering the study’s constraints of time and cost, the above said sampling scheme was more feasible than probability sampling. Despite limitations, the findings of the present survey have important implications for the management of house flies, particularly in rural environments.

## Conclusion

House flies are of major public health concerns in developing countries, particularly in the communities residing in and/or around dairy facilities. However, there is little or no information concerning community awareness of the problems associated with house flies and their management. In conclusion, education level and knowledge of house flies breeding sites in our study have a positive and significant effect on the adoption of house flies prevention practices, whereas knowledge of the problems associated with house flies and preventive measures have no effect on house flies prevention practices. The findings of this ethnoentomological survey based on local knowledge suggest that the government or health officials, keeping in view the economic importance of this notorious pest, should start health education and hygiene promotion programs for dairy farmers with special emphasis on practical ways to manage house fly populations, especially on how to manage farm yard manure. As the majority of the respondents did not have latrines in their compounds, therefore, the installation of cost effective latrine techniques with a concealed sewerage system should be the part of this program. By increasing the awareness of hygiene and its role in suppressing the spread of house flies and epidemics would increase the demands for latrines. Although knowledge of the problems associated with house flies was not directly related with house fly prevention practices, yet this is an important one because unless people have the knowledge of hazards associated with flies they would not consider it a serious pest. As we understand that there is a direct association between the farmers education level and knowledge of house flies breeding sites, and house fly prevention practices, therefore, fixing the gap between knowledge, education and management practices will remain an important task for house flies control in the future that would ultimately save many lives.

## Competing interests

The authors declare that they have no competing interests.

## Authors’ contributions

HAAK perceived and designed the study, conducted the survey, analyzed the data and wrote the manuscript. WA, SAS, UNU, MR and KZ supervised the study and helped draft the manuscript. All authors read and approved the final manuscript.
